# Photodynamic therapy for skin carcinomas: A systematic review and meta-analysis

**DOI:** 10.3389/fmed.2023.1089361

**Published:** 2023-01-19

**Authors:** Yun Ou-Yang, Yaowu Zheng, Kerry E. Mills

**Affiliations:** ^1^Department of Information, Affiliated Cancer Hospital and Institute of Guangzhou Medical University, Guangzhou, China; ^2^Guangdong Nuohui Hospital Management LLC, Guangzhou, China; ^3^Department of Science and Technology, University of Canberra, Bruce, ACT, Australia

**Keywords:** basal cell carcinoma, squamous cell carcinoma, photodynamic therapy, skin cancer, systematic review, meta-analysis

## Abstract

**Background:**

Photodynamic therapy (PDT) is increasingly used for the treatment of basal cell carcinoma (BCC) and squamous cell carcinoma (SCC). However, it is unknown whether photodynamic therapy is more effective than other commonly used treatment modalities for these cancers.

**Purpose:**

The aim of this study was to determine the relative efficacy and safety of PDT compared with placebo or other interventions for the treatment of skin carcinomas.

**Methods:**

Searches were performed in PubMed, Web of Science, Embase, and the Cochrane Central Register of Controlled Trials databases. We included randomized controlled trials comparing the PDT with other interventions in adults skin BCC or SCC that reported on lesion response, recurrence, cosmetic appearance, or safety outcomes.

**Results:**

Seventeen unique randomized controlled trials, representing 22 study arms from 21 publications were included. The included trials included 2,166 participants, comparing methyl aminolevulinic (MAL) PDT (six studies) or aminolevulinic acid (ALA) PDT (two studies). Comparators included placebo, surgery, hexaminolevulinic (HAL) PDT, erbium: yttrium-aluminum-garnet ablative factional laser (YAG-AFL) PDT, fluorouracil, and imiquimod. There were few studies available for each comparison. Mantel-Haenszel fixed effects risk ratios were calculated for response, recurrence, cosmetic outcomes, and adverse events. MAL-PDT had similar response rates to surgery, ALA-PDT, fluorouracil and imiquimod at 3- and 12 months post-intervention. The rate of recurrence was similar, showing few differences at 12 months, but at later time points (24–60 months), fewer lesions recurred with surgery and imiquimod than with PDT. PDT also caused more adverse events and pain than other interventions. However, PDT treatment was more likely to receive a “good” or “excellent” rating for cosmetic appearance than surgery or cryotherapy.

**Conclusion:**

This systematic review and meta-analysis demonstrates that the choice of treatment modality for BCC or SCC is best chosen in the context of the location and size of the lesion, the socioeconomic circumstances of the patient, as well as the patient’s preferences. We call for more high quality studies to be done, in order to enable more reliable interpretations of the data.

**Systematic review registration:**

https://www.crd.york.ac.uk/prospero/display_record.php?RecordID=368626, identifier CRD42022368626.

## Introduction

Non-melanoma skin cancers are the most commonly occurring cancer in Caucasian populations, and the incidence of both basal and squamous cell carcinomas in Europe have been increasing (1). From 1969 to 2000, 61% of the deaths associated with non-melanoma skin cancer occurred as a result of primary tumors arising on non-genital skin, and the age-adjusted mortality rate among men and women with non-genital non-melanoma skin cancer was 0.69 and 0.30 deaths per 105 at-risk individuals per year, respectively (2). For genital non-melanoma skin cancers, the rate was 0.30 per 105 at-risk individuals per year for men and 0.54 per 105 at-risk individuals per year for women (2).

The conventional treatments for non-melanoma skin cancers include surgical procedures, radiation, cryotherapy, fluorouracil, and imiquimod. Although surgery is the most common treatment for basal and squamous cell carcinomas, other less invasive methods such as fluorouracil or cryotherapy are sometimes used. Radiation may be considered as treatment for patients in whom surgery is contraindicated (3). Imiquimod has commonly been used in the treatment of various forms of basal cell carcinoma, such as nodular basal cell carcinoma and sclerodermiform basal cell carcinoma, and for various forms of squamous cell carcinoma, such as Bowen’s disease and keratoacanthoma (4, 5). Although the current treatments are effective to varying degrees, they tend to lack specificity and often do not target the tumor itself or the environment in which it exists (6). They are also associated with a high incidence of adverse effects and yield undesirable cosmetic results (7, 8). As such, alternative treatments options for patients with these conditions are needed.

Photodynamic therapy represents a safe alternative for treatment of these conditions. Photodynamic therapy uses a source of visible light to activate a photosensitizing agent (commonly aminolaevulinic acid or methyl aminolevulinic acid) applied on the skin, which releases reactive oxygen species that destroy the lesions (9). The safety and efficacy profile of photodynamic therapy in treating basal cell carcinoma and squamous cell carcinoma is well known (5). However, the relative efficacy of different modes of treatment for basal cell or squamous cell carcinomas is unknown. As such, we undertook a systematic review and meta-analysis of photodynamic therapies compared with each other, placebo, surgery, cryotherapy, imiquimod, or fluorouracil.

## Materials and methods

This systematic review and meta-analysis was performed according to the guidelines given in the Preferred Reporting Items for Systematic Review and Meta-analyses (PRISMA) (10). The protocol for this review was registered in the International Prospective Register of Systematic Review (PROSPERO) under the registration number CRD42022368626.

### Search strategy

We searched PubMed, Web of Science, Embase, and the Cochrane Library from inception to October 10, 2022. We had no limitations on the date of publication or language. The search strategy used for PubMed is given in Supplementary Table 1 and was altered for use in the other databases. The citations of included studies were searched manually to identify studies that were not returned using the search strategies.

### Inclusion/exclusion criteria

Studies were included if they met the follow criteria: randomized, controlled trial (RCT) in patients with basal cell carcinoma (BCC) or squamous cell carcinoma (SCC), who were treated with any form of photodynamic therapy (PDT), compared with another form of PDT, other therapies or placebo, followed for a period of at least 3 months in an outpatient setting. At least one of the following outcomes must have been reported: treatment success, cosmetic acceptability, pain, or adverse events.

Studies were excluded if the trial was not an RCT, the skin cancers were not BCC or SCC, if the treatments did not include PDT, if the treatment duration was too short, or if none of the required outcomes was reported. Comparisons of different protocols within a single technology (e.g., cycle number, wavelength, light source comparisons within MAL-PDT) were excluded, as were trials of recurrent cancers, and protocols that involved substantial debulking of the tumors prior to treatment. Trials adding PDT to other treatments were also excluded.

Inclusion and exclusion at the title and abstract level were carried out by two authors (YY and YZ) independently. In the case of disagreements between two authors, the article was discussed between the authors and a consensus decision was taken. After inclusion of potentially relevant abstracts, full texts of the articles were obtained and subjected to the inclusion and exclusion criteria independently by two authors (YY and YZ) using. Disagreements were resolved by consensus. Data collection was performed by one author (YY) using electronic data collection forms and extracted data was then cross checked with the articles by a second author (KM) to ensure the accuracy of the information.

### Quality assessment

The risk of bias of the included studies was carried out using the Cochrane Collaboration’s tool for assessing risk of bias in randomized trials (11). The risk of bias was assessed over seven domains: sequence generation, allocation concealment, blinding of participants and personnel, blinding of outcome assessment, incomplete outcome data, selective reporting bias, and other bias. The quality assessment was carried out by YY and checked by KM. Conflicts were resolved by consensus.

### Data extraction and data synthesis

The study characteristics and outcome data were extracted into a pre-designed Excel spreadsheet by YZ and checked by KM. Where data were reported by both the clinician and the patient (e.g., cosmetic appearance), we extracted the patient-reported data. Response rates were calculated at the end of the treatment period, regardless of the number of treatments each group received. If data were available at the patient level, we extracted this; if data were only available at the lesion level, we extracted lesion-level data.

All outcomes were reported as dichotomous data; we therefore calculated Mantel-Haenszel risk ratios with 95% confidence intervals using a random effects model. A random effects model was chosen as we expected the heterogeneity between studies to arise mostly by factors other than chance, such as differences in carcinoma size, location, or depth, treatment protocols, and differences in the populations. Where a study reported on more than two arms and both were used in a single analysis, the arms were separated into subgroups, and the overall meta-analyzed result was removed from the analysis.

Subgroup analysis was used to differentiate between the location of the lesion. Meta-regression was undertaken using type of carcinoma (SCC or BCC) and comparators as covariates. Meta-regression was carried out using OpenMetaAnalyst (12) on outcomes that had nine or more study arms comparing any type of PDT with a non-PDT comparator.

## Results

### Studies

**FIGURE 1:**
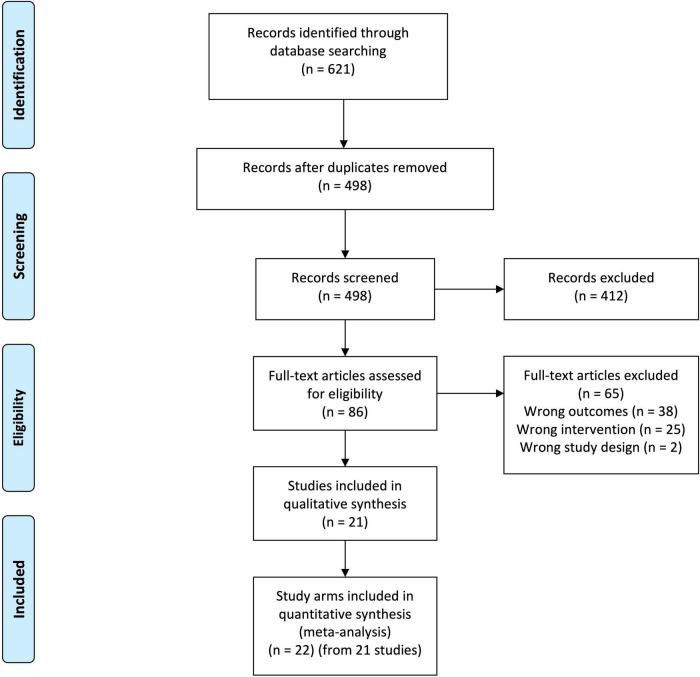
Preferred reporting items for systematic review and meta-analyses (PRISMA) diagram. From 621 records identified from database searching, 123 were duplicates. Screening of these records at the title and abstract level resulted in the exclusion of 412 records. The 86 remaining records were obtained as full texts and submitted to inclusion and exclusion. This resulted in the exclusion of 65 articles. Twenty-one articles representing 22 study arms from 17 separate randomized controlled trials were included in the final analysis.

The database searches identified 621 citations, of which 123 were duplicates (Figure 1). The remaining 498 citations were subjected to inclusion and exclusion at the abstract level. This resulted in the exclusion of 412 abstracts. The remaining full texts for the remaining 86 abstracts were sourced and subjected to the inclusion and exclusion criteria. Of these, 21 publications reporting on 22 study arms from 17 individual RCTs were included (13–33).

The included studies involved 2,166 participants (Table 1). Twelve trials were in people with basal cell carcinomas, and five trials involved people with squamous cell carcinomas. Most studies used methyl aminolevulinic acid (MAL) as the photosensitizing agent in PDT. These studies compared MAL-PDT with aminolevulinic acid (ALA) PDT, erbium: yttrium-aluminum-garnet ablative fractional laser-assisted (YAG-AFL) PDT, hexaminolevulinic (HAL) PDT, surgical excision, cryotherapy, imiquimod, fluorouracil, and placebo. Five studies compared ALA-PDT with surgical excision, cryotherapy, and fluorouracil. The mean age of the participants was 67.3 years, and on average 49.7% of participants were female.

**TABLE 1:** Characteristics of included studies.

Study ID	Authors	Clinical trial identifier	Carcinoma type	PDT-n	Control-n	PDT type	Control	Follow up times	Average/Median age	% female
EudraCT-2013-003241-42	Morton 2018	EudraCT-2013-003241-42	BCC	110	121	MAL-PDT	ALA-PDT	3 m, 12 m	67	43.3
ISRCTN 79701845	Arits 2013/1	ISRCTN 79701845	BCC	202	198	MAL-PDT	Imiquimod	3 m, 12 m	63	50.5
ISRCTN 79701845	Arits 2013/2	ISRCTN 79701845	BCC	202	201	MAL-PDT	Fluorouracil	3 m, 12 m	63	48.0
ISRCTN 79701845	Jansen 2018	ISRCTN 79701845	BCC	153	157	MAL-PDT	Fluorouracil	5 y	63	48.0
ISRCTN 79701845	Jansen 2018	ISRCTN 79701845	BCC	153	148	MAL-PDT	Imiquimod	5 y	63	50.5
ISRCTN 79701845	Roozeboom 2014	ISRCTN 79701845	BCC	202	198	MAL-PDT	Imiquimod	12 m	63	50.6
ISRCTN 79701845	Roozeboom 2016/1	ISRCTN 79701845	BCC	202	198	MAL-PDT	Imiquimod	36 m	63	50.6
ISRCTN 79701845	Roozeboom 2016/2	ISRCTN 79701845	BCC	202	201	MAL-PDT	Fluorouracil	36 m	63	48.0
Basset-Seguin 2008	Basset-Seguin 2008	N/A	BCC	66	58	MAL-PDT	Cryotherapy	5 y		
Berroeta 2007	Berroeta 2007	N/A	BCC	21	19	ALA-PDT	Surgery	3 m, 6 m, 12 m	72	38.7
Foley 2009	Foley 2009	N/A	BCC	66	65	MAL-PDT	Placebo	3 m	66	24.4
Ko 2013	Ko 2013	N/A	SCC	19	19	MAL-PDT	YAG-AFL-PDT	3 m, 12 m	52.4	39
Morton 1996	Morton 1996	N/A	SCC	20	20	ALA-PDT	Cryotherapy	3 m, 12 m	76	84.0
Morton 2006/1	Morton 2006/1	N/A	SCC	96	17	MAL-PDT	Placebo	3 m, 12 m	72	62.8
Morton 2006/2	Morton 2006/2	N/A	SCC	96	82	MAL-PDT	Cryotherapy	3 m, 12 m	73	60.7
Morton 2006/3	Morton 2006/3	N/A	SCC	96	30	MAL-PDT	Fluorouracil	3 m, 12 m	72	62.7
Mosterd 2008	Mosterd 2008	N/A	BCC	83	88	ALA-PDT	Surgery	3 m, 36 m	65	49.7
Rhodes 2004	Rhodes 2004	N/A	BCC	52	49	MAL-PDT	Surgery	12 m, 24 m	68	39.6
Rhodes 2004 FU	Rhodes 2007	N/A	BCC	52	49	MAL-PDT	Surgery	5 y	68	39.6
Salim 2003	Salim 2003	N/A	SCC	20	20	ALA-PDT	Fluorouracil	3 m, 12 m	76	80.0
Szeimie 2008	Szeimie 2008	N/A	BCC	100	96	MAL-PDT	Surgery	3 m, 12 m	64	33.7
Wang 2001	Wang 2001	N/A	BCC	47	41	ALA-PDT	Cryotherapy	12 m	NR	50.0
NCT01491711	Kessels 2017	NCT01491711	BCC	80	82	MAL-PDT	ALA-PDT	3 m, 12 m	65	53.7
NCT02018679	Choi 2016	NCT02018679	BCC	19	20	MAL-PDT	YAG-AFL-PDT	3 m, 12 m	65	45.9
NCT02367547	Salmivuori 2020	NCT02367547	BCC	31	33	MAL-PDT	ALA-PDT	3 m	72	28.3
NCT02367547	Salmivuori 2020	NCT02367547	BCC	31	31	MAL-PDT	HAL-PDT	3 m	72	43.1
NCT02666534	Choi 2017	NCT02666534	SCC	24	21	MAL-PDT	YAG-AFL-PDT	3 m, 12 m, 24 m	76	62.2

ALA-PDT, aminolevulinic acid photodynamic therapy; BCC, basal cell carcinoma; HAL-PDT, hexaminolevulinic acid photodynamic therapy; m, months; MAL-PDT, methyl aminolevulinic acid photodynamic therapy; N/A, not applicable; NR, not reported; PDT, photodynamic therapy; y, years; YAG-AFL-PDT, erbium: yttrium-aluminum-garnet ablative factional laser photodynamic therapy.

Study quality as determined by the Cochrane Collaboration’s tool for the assessment of risk of bias in interventions studies was generally good (Supplementary Figure 1). However, due to the nature of the interventions, it was often impossible to blind the participants to their intervention. To compensate for this, the outcome assessors were frequently blinded to the allocation of the patients they assessed. Five of the clinical trials were funded by companies with a financial interest in the outcomes; these trials usually had at least one company employee on the author list of the publication(s).

### Response

**FIGURE 2:**
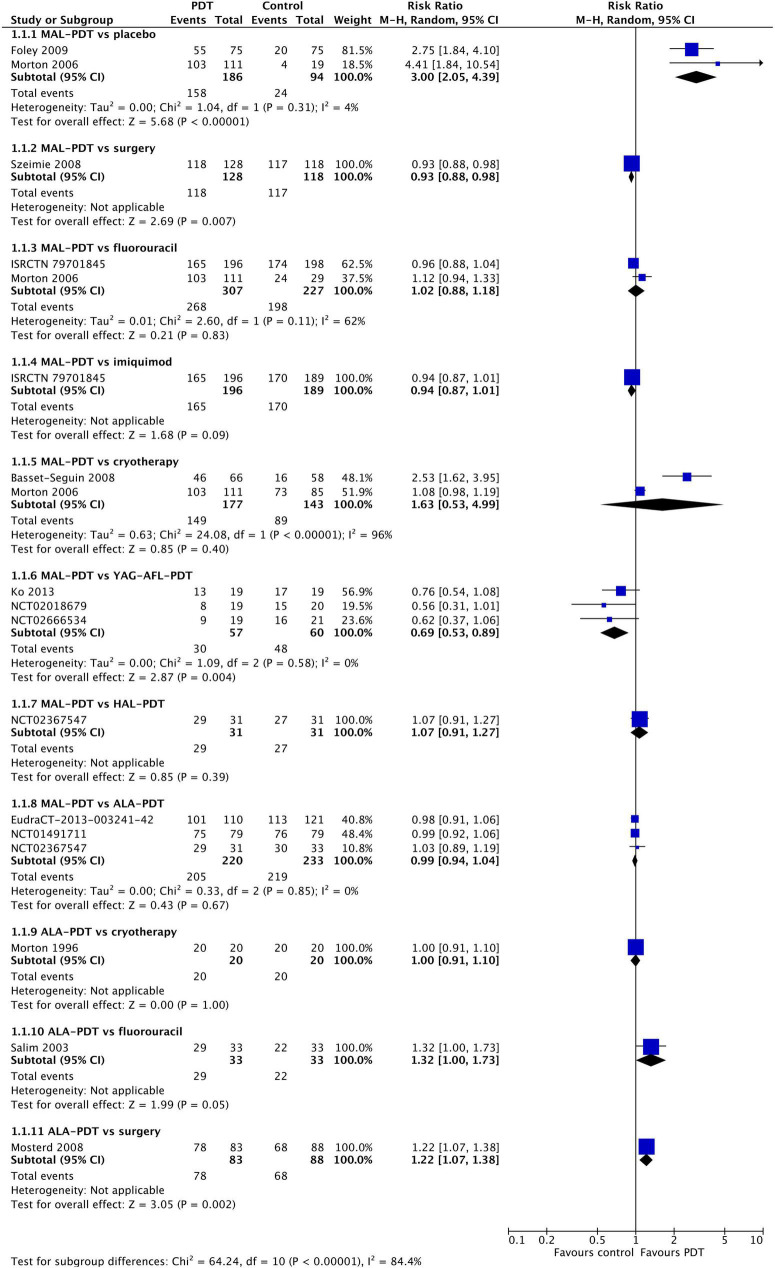
Meta-analysis of the risk of response at 3 months post-treatment by comparator. Data are risk ratios with 95% confidence intervals.

All clinical trials reported on response at 3 months post-treatment (Figure 2). MAL-PDT was significantly superior to placebo (two studies; RR: 3.00 (95% CI: 2.05 to 4.39); *P* < 0.00001), but statistically less effective than surgery (one study) and YAG-AFL-PDT (three studies). No other comparisons were statistically significant.

**FIGURE 3:**
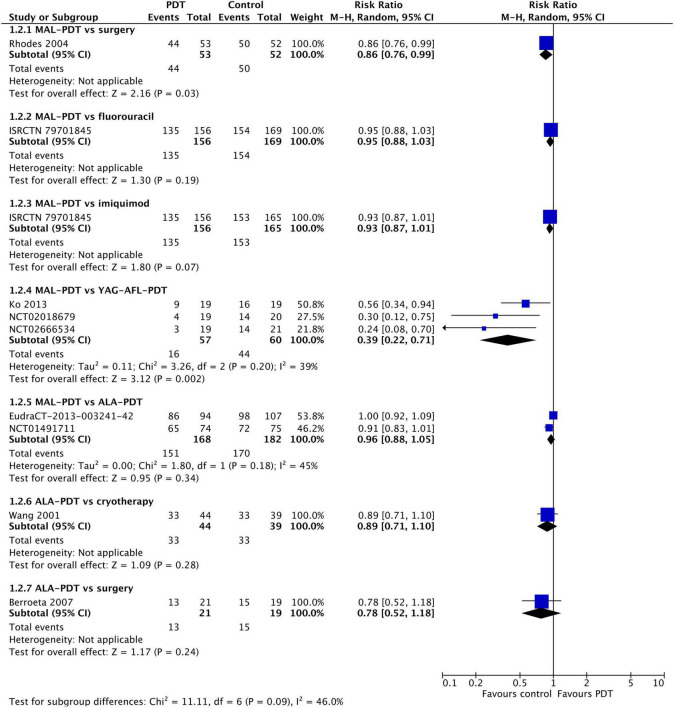
Meta-analysis of the risk of response at 12 months post-treatment by comparator. Data are risk ratios with 95% confidence intervals.

At 12 months post-treatment, similar results were observed (Figure 3). MAL-PDT was less effective than surgery (one study) and YAG-AFL-PDT (three studies). At 24–60 months post-intervention (Supplementary Figure 2), MAL-PDT was statistically inferior to surgery and YAG-AFL-PDT at 24 m (one study), and imiquimod at 36 m (one study).

Analyses by location of the original lesion found few differences at 3 months (Supplementary Figure 3). MAL-PDT was more effective than placebo on the extremities, but less effective than surgery on the neck/trunk. At 12 months, imiquimod was statistically superior to MAL-PDT for lesions on the trunk, whereas MAL-PDT was superior to imiquimod for lesions on the extremities (Supplementary Figure 4). However, each of these findings resulted from a single trial, so interpretations should be made with caution.

Meta-regression of all active control studies revealed no significant differences between imiquimod, cryotherapy, fluorouracil, and surgery (Supplementary Table 2). We therefore undertook a meta-regression of all active control studies by type of carcinoma (Supplementary Table 3). There were no significant differences between basal cell and squamous cell carcinomas for this outcome, suggesting that the treatments are similarly effective for both carcinoma types.

### Recurrence

**FIGURE 4:**
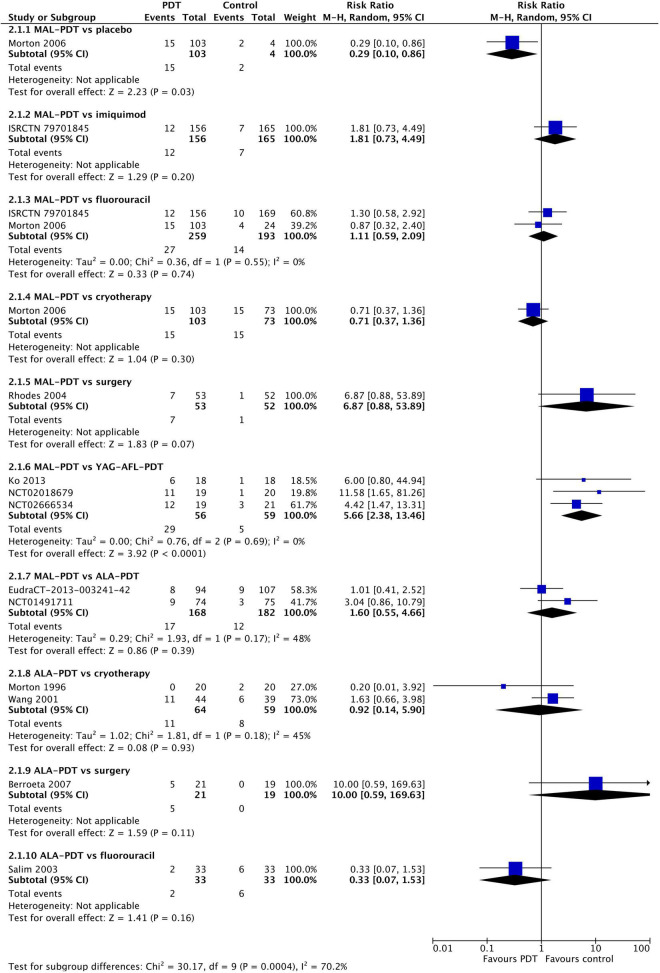
Meta-analysis of the risk of recurrence at 12 months post-treatment by comparator. Data are risk ratios with 95% confidence intervals.

**FIGURE 5:**
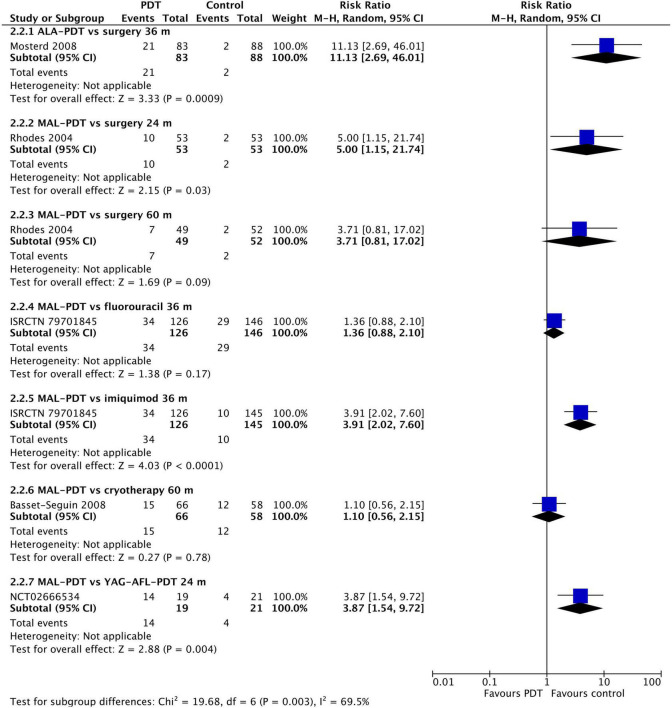
Meta-analysis of the risk of recurrence at 24 to 60 months post-treatment by comparator. Data are risk ratios with 95% confidence intervals.

At 12 months post-intervention, MAL-PDT was more effective than placebo (one study), but YAG-AFL-PDT was more effective than MAL-PDT (three studies; Figure 4). The risk of recurrence after MAL-PDT was 5.66 times (95% CI: 2.38, 13.46) that of YAG-AFL-PDT (*P* < 0.00001). At 24 months, this increased risk of recurrence remained (one study per comparison; Figure 5). In addition, both MAL-PDT and ALA-PDT were inferior to surgery at 24- and 36-month post-intervention, respectively, and MAL-PDT was inferior to YAG-AFL-PDT and imiquimod at 24- and 36-month post-intervention, however there was only one study per comparison, so these results should be interpreted with caution.

Analyses by location of the original lesion showed few differences (Supplementary Figure 5). MAL-PDT was superior to cryotherapy on the face/scalp, superior to placebo on the neck/trunk, and superior to imiquimod on the extremities. In contrast, imiquimod was significantly less likely than MAL-PDT to result in lesion recurrence on the body trunk. However, each of these findings resulted from a single trial, so interpretations should be made with caution. There were too few studies to undertake meta-regression.

### Cosmetic outcomes

**FIGURE 6:**
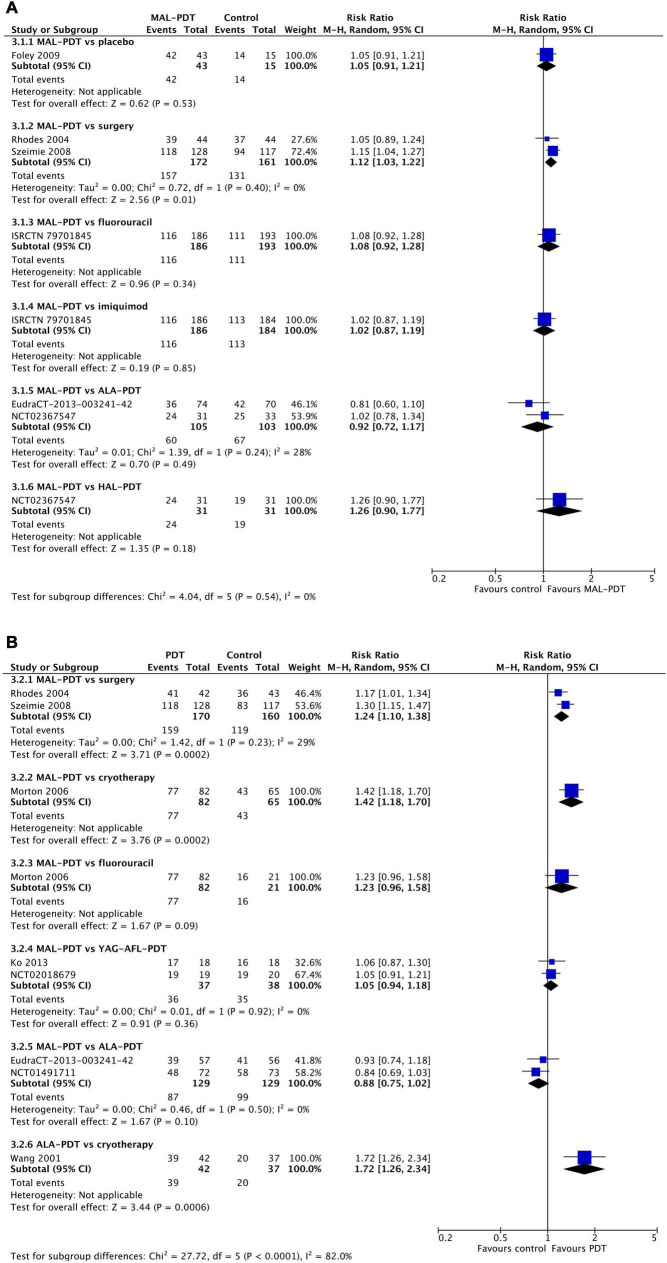
Meta-analysis of the risk of a cosmetic rating of “good” or “excellent” at 3 months **(A)** or 12 months **(B)** post-intervention by comparator. Data are risk ratios with 95% confidence intervals.

After 3 months, there were no differences in the chance of good/excellent ratings for cosmetic appearance of lesions between MAL-PDT and ALA-PDT (two studies), HAL-PDT, imiquimod, fluorouracil, and placebo (one study per comparison; Figure 6A). The only exception to this was the comparison of MAL-PDT with surgery, which significantly favored MAL-PDT (two studies; RR: 1.12 (95% CI: 1.03, 1.22); *p* = 0.01). At 12 months, ALA-PDT showed superiority over cryotherapy (one study), and MAL-PDT was superior to surgery (two studies) and cryotherapy (one study) for this outcome (Figure 6B). There were too few studies to undertake meta-regression.

### Adverse events

**FIGURE 7:**
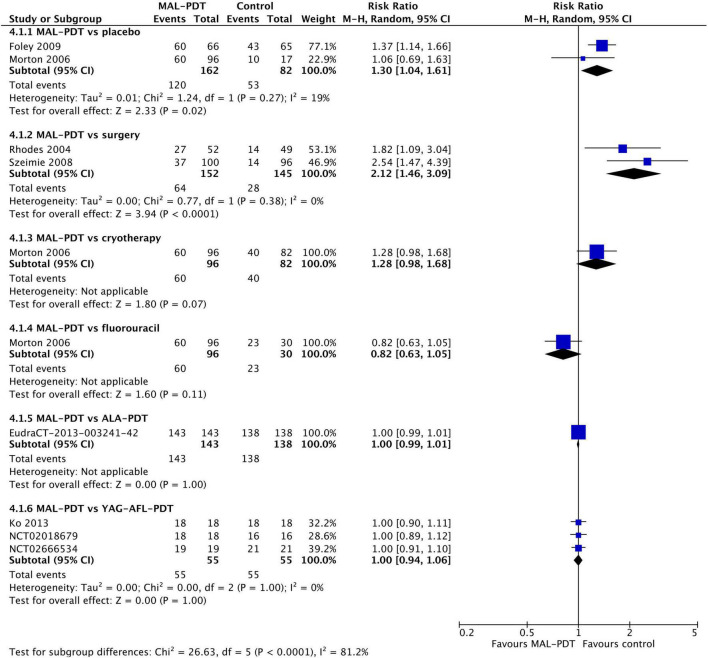
Meta-analysis of the risk of adverse events by comparator. Data are risk ratios with 95% confidence intervals.

The rates of any adverse event were relatively high in all studies (Figure 7). There were no significant differences between the different forms of PDT (MAL vs. ALA (one study) and MAL vs. YAG-AFL (three studies)). However, MAL-PDT caused significantly more adverse events than surgery (two studies; RR: 2.12; 95% CI: 1.46 to 3.09; *P* < 0.0001).

**FIGURE 8:**
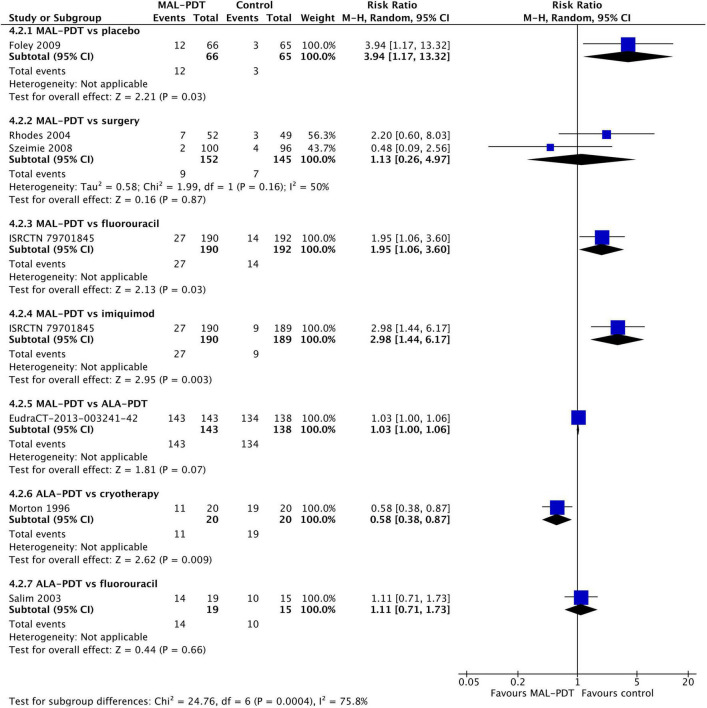
Meta-analysis of the risk of pain during the procedure by comparator. Data are risk ratios with 95% confidence intervals.

The incidence of pain during the procedure was higher in people who had MAL-PDT than imiquimod, fluorouracil, or placebo (one study per comparison; Figure 8), and lower in those undergoing ALA-PDT than cryotherapy (one study). There was no difference in the incidence of pain between MAL-PDT and ALA-PDT (one study), between ALA-PDT and fluorouracil (one study), and between MAL-PDT and surgery (two studies). There were too few studies to undertake meta-regression. Peak pain severity showed a slightly different picture. The only statistically significant difference in pain intensity was between ALA-PDT and surgery (one study; Supplementary Figure 6).

### Publication bias

The potential for publication bias was examined by visual examination of funnel plots produced for response, recurrence, cosmetic outcomes, and adverse events (Supplementary Figure 7). All plots appeared symmetrical, with little obvious skew in data. We attempted to undertake an analysis of bias by comparing industry-funded with government/university funded studies (Supplementary Figure 8). We used studies reporting on response at 3 months for comparisons that showed a statistically significant advantage toward PDT in the overall meta-analysis. We found no statistically significant differences between the two subgroups, however, heterogeneity was extremely high (96 and 85% for industry-funded and non-industry-funded, respectively). This heterogeneity was likely a result of combing different control types.

## Discussion

Our systematic review and meta-analysis demonstrates for the first time the evidence for the relative efficacy and safety of PDT compared with other potential treatments. We found that MAL-PDT was superior to placebo, and ALA-PDT was superior to surgery, but PDT, surgery, fluorouracil, and imiquimod have similar rates of response and recurrence over the short and medium term. However, over the longer term (24–60 m), PDT-treated lesions are significantly more likely to recur than those that were surgically resected, or those treated with imiquimod. PDT is also more likely to cause intra-procedural pain and adverse events. However, PDT is more likely to be rated “good” or “excellent” compared with cryotherapy and surgery. Given the small number of available studies, the choice of treatment for any particular lesion is best left to the discretion of the treating physician, taking into account the individual patient’s preferences.

Interestingly, differences between the photosensitizing agents used with PDT emerged. Whereas MAL-PDT was significantly less likely than surgery to result in response of a lesion at 3 months (32), ALA-DT was significantly more likely than surgery to result in response (25). Whether these differences are real and robust, however, is unclear, as Szeimie et al. (32) treated small superficial lesions, whereas Mosterd et al. (25) treated nodular BCCs. Furthermore, three head-to-head trials failed to show any differences between MAL-PDT and ALA-PDT (20, 24, 31). Intriguingly, the meta-analysis indicates that YAG-AFL-PDT may be superior to MAL-PDT for response and recurrence, without compromising on cosmetic outcomes or risk of adverse events (16, 17, 21). However, larger studies will be required to confirm this result.

Although cosmetic concerns are sometimes minimized or dismissed, they can have serious effects on patients. Patients with visible and unpleasant scarring can suffer from embarrassment, isolation and a modification of social activities (34), along with self-consciousness, unhappiness and insecurity (35). Nevertheless, excision, especially of melanomas and SCCs, does lead to an increase in quality of life, probably due to a reduction in the anxiety associated with a diagnosis of skin cancer (34). It is thus vital that the location of the lesion, the likely size and visibility of the resulting scar, and the personal circumstances, activities, and preferences of the individual patient are considered when choosing the treatment for any particular lesion.

## Conclusion

Basal cell carcinoma and SCC lesions cause significant economic burdens for health organizations all around the world. This disease also leads to chronic discomfort and a reduction in quality of life for patients, as it can cause poor cosmetic effects. Finding an appropriate method for treatment of these lesions has remained a challenge as the number of treatment methods increases. Our results suggest that PDT is can be an effective method for treatment of these lesions. However, further studies are required before any strong recommendations can be made.

## Data availability statement

The raw data supporting the conclusions of this article will be made available by the authors, without undue reservation.

## Author contributions

YO-Y and YZ did the database searching, citation management, and inclusion and exclusion of records. YZ did the data extraction, study quality assessment, which was checked by KM. KM did the meta-analysis and meta-regression in consultation with YO-Y and YZ. KM wrote the manuscript in consultation with YO-Y and YZ. All authors contributed to the article and approved the submitted version.
