# Supplementary material for: Photodynamic therapy for skin carcinomas: A systematic review and meta-analysis
Source: Front Med (Lausanne). 2023 Jan 19;10:1089361. doi: 10.3389/fmed.2023.1089361 (PMC9892842; doi:10.3389/fmed.2023.1089361)
Supplement: Supplementary file 1 [file Data_Sheet_1.PDF]

Photodynamic therapy for skin carcinomas: a systematic review and meta-analysis – supplementary materials

Yun Ou Yang[1], Yaowu Zheng[2], Kerry E Mills[3*]

[1]Department of Information, Affiliate Cancer Hospital & Institute of Guangzhou Medical University, Guangzhou, P.R. China

[2]Guangdong Nuohui Hospital Management LLC, Guangzhou, P.R. China

[3]Department of Science and Technology, University of Canberra, Bruce, ACT, Australia

Supplementary tables

Supplementary table 1: Search strategy

No.	Connector	Search term
1		basal cell carcinoma OR squamous cell carcinoma OR BCC OR SCC OR Bowen OR skin cancer [Title/Abstract]
2	AND	photodynamic therapy OR PDT [Title/Abstract]
3	AND	randomized clinical trial [Publication type]

Supplementary table 2: meta-regression of active control studies by control type

Covariate	Level	Studies	Coefficients	Lower CI	Upper CI	Std Error	p-Value
Intercept			-0.066	-0.307	0.174	0.123	0.589
Control	Imiquimod	1					
	Cryotherapy	3	0.201	-0.090	0.493	0.149	0.176
	Fluorouracil	3	0.143	-0.148	0.433	0.148	0.337
	Surgery	2	0.113	-0.183	0.410	0.151	0.454

Supplementary table 3: meta-regression of active control studies by carcinoma type

Covariate	Level	Studies	Coefficients	Lower CI	Upper CI	Std Error	p-Value
Intercept			0.049	-0.072	0.170	0.062	0.426
Control	BCC	5					
	SCC	4	0.043	-0.142	0.228	0.094	0.646

Supplementary figures

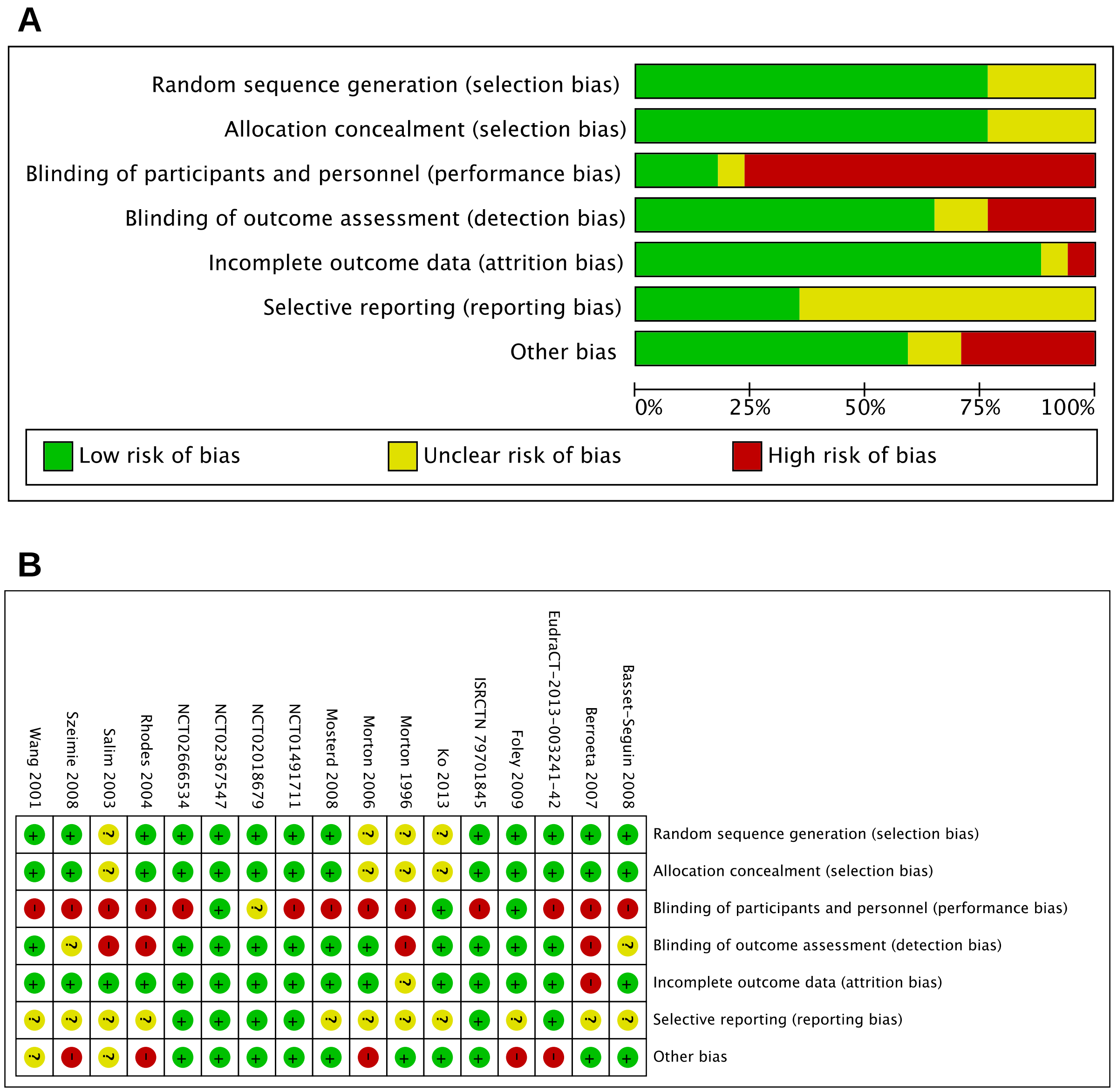

Supplementary figure 1: Risk of bias graph (A) and summary (B) of included clinical trials. Risk of bias was identified over seven domains: random sequence generation, allocation concealment, blinding of participants and personnel, blinding of outcome assessment, incomplete outcome data, selective reporting, and other bias.

Study or Subgroup	PDT Events	PDT Total	Control Events	Control Total	Weight	Risk Ratio M-H, Random, 95% CI
**1.3.1 MAL-PDT vs surgery 24 m**						
Rhodes 2004	32	53	44	53	100.0%	0.73 [0.57, 0.93]
**Subtotal (95% CI)**		**53**		**53**	**100.0%**	**0.73 [0.57, 0.93]**
Total events	32		44			
Heterogeneity: Not applicable						
Test for overall effect: Z = 2.50 (P = 0.01)						
**1.3.2 MAL-PDT vs surgery 60m**						
Rhodes 2004	42	49	50	52	100.0%	0.89 [0.79, 1.01]
**Subtotal (95% CI)**		**49**		**52**	**100.0%**	**0.89 [0.79, 1.01]**
Total events	42		50			
Heterogeneity: Not applicable						
Test for overall effect: Z = 1.78 (P = 0.08)						
**1.3.3 MAL-PDT vs fluorouracil 36 m**						
ISRCTN 79701845	92	126	117	146	100.0%	0.91 [0.80, 1.04]
**Subtotal (95% CI)**		**126**		**146**	**100.0%**	**0.91 [0.80, 1.04]**
Total events	92		117			
Heterogeneity: Not applicable						
Test for overall effect: Z = 1.37 (P = 0.17)						
**1.3.4 MAL-PDT vs imiquimod 36 m**						
ISRCTN 79701845	92	126	135	145	100.0%	0.78 [0.70, 0.88]
**Subtotal (95% CI)**		**126**		**145**	**100.0%**	**0.78 [0.70, 0.88]**
Total events	92		135			
Heterogeneity: Not applicable						
Test for overall effect: Z = 4.14 (P < 0.0001)						
**1.3.5 MAL-PDT vs YAG-AFL-PDT 24 m**						
NCT02666534	2	19	13	21	100.0%	0.17 [0.04, 0.66]
**Subtotal (95% CI)**		**19**		**21**	**100.0%**	**0.17 [0.04, 0.66]**
Total events	2		13			
Heterogeneity: Not applicable						
Test for overall effect: Z = 2.57 (P = 0.01)						

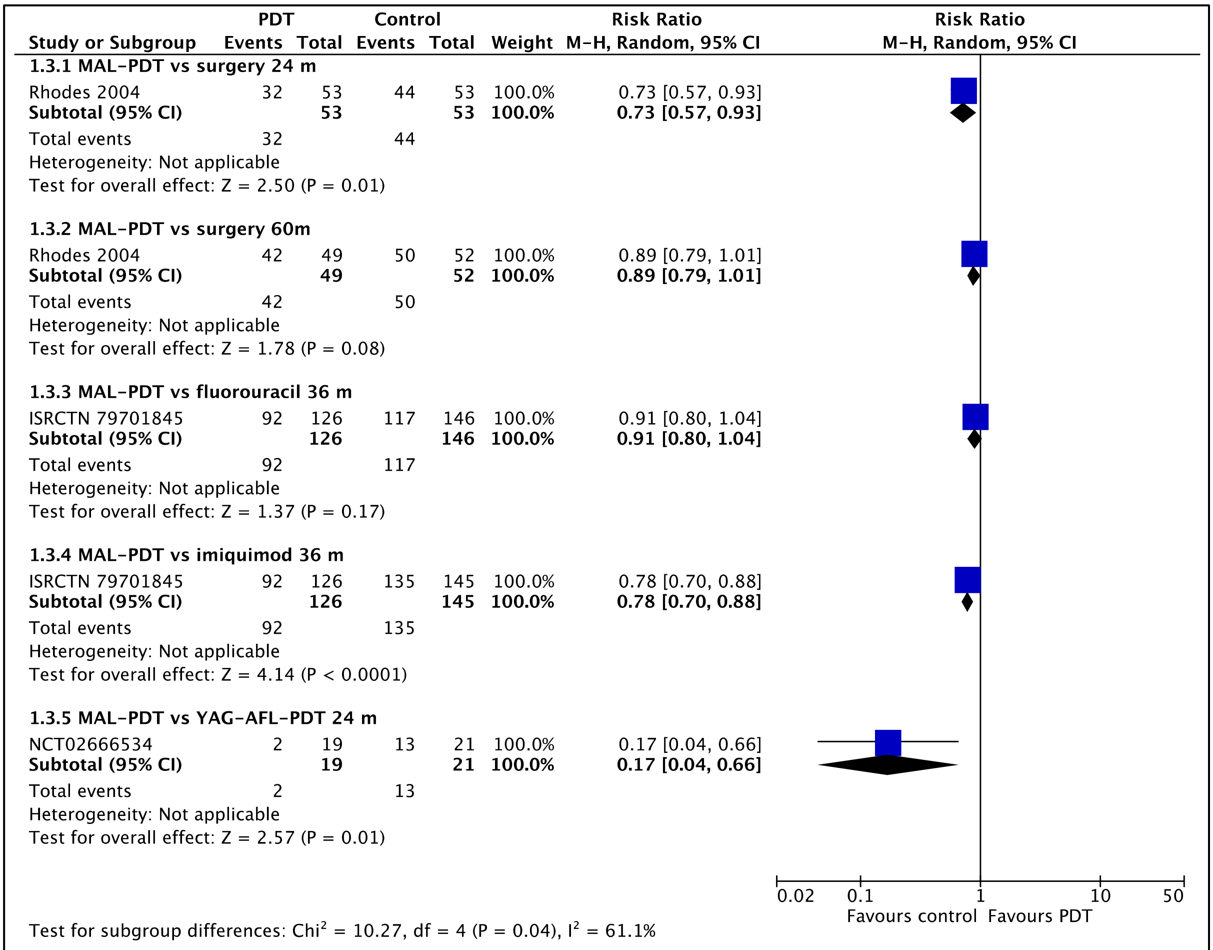

Test for subgroup differences: $Chi^2 = 10.27$, df = 4 (P = 0.04), $I^2 = 61.1\%$

Supplementary figure 2: meta-analysis of response at 24–60 months by control type. Data are risk ratios with 95% confidence intervals.

Study or Subgroup	PDT Events	PDT Total	Control Events	Control Total	Weight	Risk Ratio M-H, Random, 95% CI
**1.4.1 MAL-PDT vs placebo face/scalp**						
Morton 2006	27	28	2	5	0.6%	2.41 [0.82, 7.07]
**Subtotal (95% CI)**		**28**		**5**	**0.6%**	**2.41 [0.82, 7.07]**
Total events	27		2			
Heterogeneity: Not applicable						
Test for overall effect: Z = 1.60 (P = 0.11)						
**1.4.2 MAL-PDT vs surgery face/scalp**						
Szeimie 2008	15	15	4	4	5.0%	1.00 [0.74, 1.36]
**Subtotal (95% CI)**		**15**		**4**	**5.0%**	**1.00 [0.74, 1.36]**
Total events	15		4			
Heterogeneity: Not applicable						
Test for overall effect: Z = 0.00 (P = 1.00)						
**1.4.3 MAL-PDT vs cryotherapy face/scalp**						
Morton 2006	27	28	17	21	7.4%	1.19 [0.96, 1.48]
**Subtotal (95% CI)**		**28**		**21**	**7.4%**	**1.19 [0.96, 1.48]**
Total events	27		17			
Heterogeneity: Not applicable						
Test for overall effect: Z = 1.56 (P = 0.12)						
**1.4.4 MAL-PDT vs flurouracil face/scalp**						
Morton 2006	27	28	5	6	3.9%	1.16 [0.80, 1.67]
**Subtotal (95% CI)**		**28**		**6**	**3.9%**	**1.16 [0.80, 1.67]**
Total events	27		5			
Heterogeneity: Not applicable						
Test for overall effect: Z = 0.78 (P = 0.43)						
**1.4.5 MAL-PDT vs ALA-PDT face/scalp**						
EudraCT-2013-003241-42	12	17	14	17	3.7%	0.86 [0.59, 1.25]
**Subtotal (95% CI)**		**17**		**17**	**3.7%**	**0.86 [0.59, 1.25]**
Total events	12		14			
Heterogeneity: Not applicable						
Test for overall effect: Z = 0.80 (P = 0.42)						
**1.4.6 MAL-PDT vs placebo neck/trunk**						
Morton 2006	13	14	1	2	0.4%	1.86 [0.46, 7.48]
**Subtotal (95% CI)**		**14**		**2**	**0.4%**	**1.86 [0.46, 7.48]**
Total events	13		1			
Heterogeneity: Not applicable						
Test for overall effect: Z = 0.87 (P = 0.38)						
**1.4.7 MAL-PDT vs surgery neck/trunk**						
Szeimie 2008	69	76	82	83	13.4%	0.92 [0.85, 0.99]
**Subtotal (95% CI)**		**76**		**83**	**13.4%**	**0.92 [0.85, 0.99]**
Total events	69		82			
Heterogeneity: Not applicable						
Test for overall effect: Z = 2.20 (P = 0.03)						
**1.4.8 MAL-PDT vs cryotherapy neck/trunk**						
Morton 2006	13	14	13	13	8.2%	0.93 [0.77, 1.14]
**Subtotal (95% CI)**		**14**		**13**	**8.2%**	**0.93 [0.77, 1.14]**
Total events	13		13			
Heterogeneity: Not applicable						
Test for overall effect: Z = 0.69 (P = 0.49)						
**1.4.9 MAL-PDT vs flurouracil neck/trunk**						
Morton 2006	13	14	2	2	2.1%	1.08 [0.63, 1.84]
**Subtotal (95% CI)**		**14**		**2**	**2.1%**	**1.08 [0.63, 1.84]**
Total events	13		2			
Heterogeneity: Not applicable						
Test for overall effect: Z = 0.28 (P = 0.78)						
**1.4.10 MAL-PDT vs ALA-PDT neck/trunk**						
EudraCT-2013-003241-42	84	87	95	97	14.3%	0.99 [0.94, 1.04]
**Subtotal (95% CI)**		**87**		**97**	**14.3%**	**0.99 [0.94, 1.04]**
Total events	84		95			
Heterogeneity: Not applicable						
Test for overall effect: Z = 0.57 (P = 0.57)						
**1.4.11 MAL-PDT vs placebo extremities**						
Morton 2006	63	69	1	12	0.2%	10.96 [1.68, 71.66]
**Subtotal (95% CI)**		**69**		**12**	**0.2%**	**10.96 [1.68, 71.66]**
Total events	63		1			
Heterogeneity: Not applicable						
Test for overall effect: Z = 2.50 (P = 0.01)						
**1.4.12 MAL-PDT vs surgery extremities**						
Szeimie 2008	34	37	31	31	11.9%	0.92 [0.83, 1.03]
**Subtotal (95% CI)**		**37**		**31**	**11.9%**	**0.92 [0.83, 1.03]**
Total events	34		31			
Heterogeneity: Not applicable						
Test for overall effect: Z = 1.44 (P = 0.15)						
**1.4.13 MAL-PDT vs cryotherapy extremities**						
Morton 2006	63	69	43	51	10.6%	1.08 [0.94, 1.24]
**Subtotal (95% CI)**		**69**		**51**	**10.6%**	**1.08 [0.94, 1.24]**
Total events	63		43			
Heterogeneity: Not applicable						
Test for overall effect: Z = 1.12 (P = 0.26)						
**1.4.14 MAL-PDT vs flurouracil extremities**						
Morton 2006	63	69	17	21	7.4%	1.13 [0.91, 1.41]
**Subtotal (95% CI)**		**69**		**21**	**7.4%**	**1.13 [0.91, 1.41]**
Total events	63		17			
Heterogeneity: Not applicable						
Test for overall effect: Z = 1.07 (P = 0.28)						
**1.4.15 MAL-PDT vs ALA-PDT extremities**						
EudraCT-2013-003241-42	22	23	31	34	10.8%	1.05 [0.92, 1.20]
**Subtotal (95% CI)**		**23**		**34**	**10.8%**	**1.05 [0.92, 1.20]**
Total events	22		31			
Heterogeneity: Not applicable						
Test for overall effect: Z = 0.69 (P = 0.49)						
**Total (95% CI)**		**588**		**399**	**100.0%**	**1.02 [0.94, 1.11]**
Total events	545		358			
Heterogeneity: Tau^2 = 0.01; Chi^2 = 43.63, df = 14 (P < 0.0001); I^2 = 68%						
Test for overall effect: Z = 0.48 (P = 0.63)						
Test for subgroup differences: Chi^2 = 23.02, df = 14 (P = 0.06), I^2 = 39.2%						

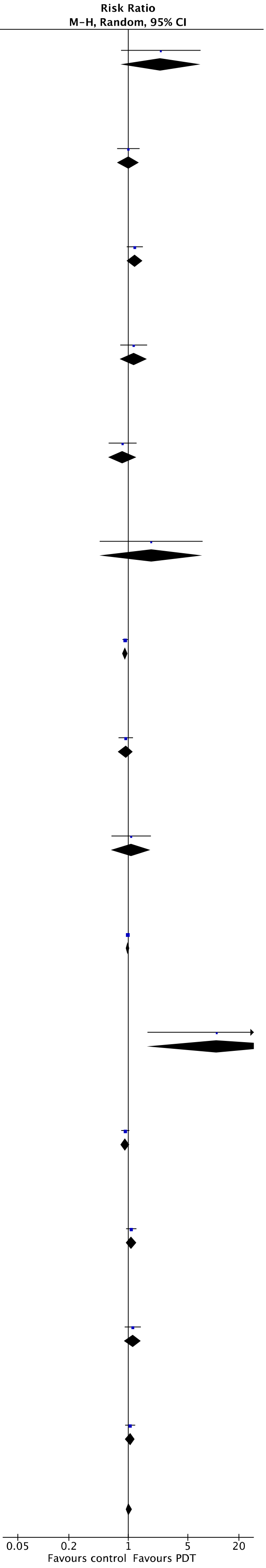

Supplementary figure 3: meta-analysis of response at 3 months by control type and location of lesion. Data are risk ratios with 95% CIs.

Study or Subgroup	PDT Events	PDT Total	Control Events	Control Total	Weight	Risk Ratio M-H, Random, 95% CI
**1.5.1 MAL-PDT vs ALA-PDT face/scalp**						
EudraCT-2013-003241-42	9	11	12	13	100.0%	0.89 [0.64, 1.22]
**Subtotal (95% CI)**		**11**		**13**	**100.0%**	**0.89 [0.64, 1.22]**
Total events	9		12			
Heterogeneity: Not applicable						
Test for overall effect: Z = 0.74 (P = 0.46)						
**1.5.2 MAL-PDT vs imiquimod head/neck**						
ISRCTN 79701845	15	24	16	20	100.0%	0.78 [0.53, 1.14]
**Subtotal (95% CI)**		**24**		**20**	**100.0%**	**0.78 [0.53, 1.14]**
Total events	15		16			
Heterogeneity: Not applicable						
Test for overall effect: Z = 1.27 (P = 0.20)						
**1.5.3 MAL-PDT vs ALA-PDT neck/trunk**						
EudraCT-2013-003241-42	73	79	84	90	100.0%	0.99 [0.91, 1.08]
**Subtotal (95% CI)**		**79**		**90**	**100.0%**	**0.99 [0.91, 1.08]**
Total events	73		84			
Heterogeneity: Not applicable						
Test for overall effect: Z = 0.23 (P = 0.82)						
**1.5.4 MAL-PDT vs imiquimod trunk**						
ISRCTN 79701845	79	115	104	116	100.0%	0.77 [0.67, 0.88]
**Subtotal (95% CI)**		**115**		**116**	**100.0%**	**0.77 [0.67, 0.88]**
Total events	79		104			
Heterogeneity: Not applicable						
Test for overall effect: Z = 3.78 (P = 0.0002)						
**1.5.5 MAL-PDT vs ALA-PDT extremities**						
EudraCT-2013-003241-42	19	20	29	31	100.0%	1.02 [0.89, 1.16]
**Subtotal (95% CI)**		**20**		**31**	**100.0%**	**1.02 [0.89, 1.16]**
Total events	19		29			
Heterogeneity: Not applicable						
Test for overall effect: Z = 0.22 (P = 0.83)						
**1.5.6 MAL-PDT vs imiquimod extremities**						
ISRCTN 79701845	50	57	38	53	100.0%	1.22 [1.01, 1.49]
**Subtotal (95% CI)**		**57**		**53**	**100.0%**	**1.22 [1.01, 1.49]**
Total events	50		38			
Heterogeneity: Not applicable						
Test for overall effect: Z = 2.03 (P = 0.04)						

Test for subgroup differences: $Chi^2 = 18.73$, df = 5 (P = 0.002), $I^2 = 73.3\%$

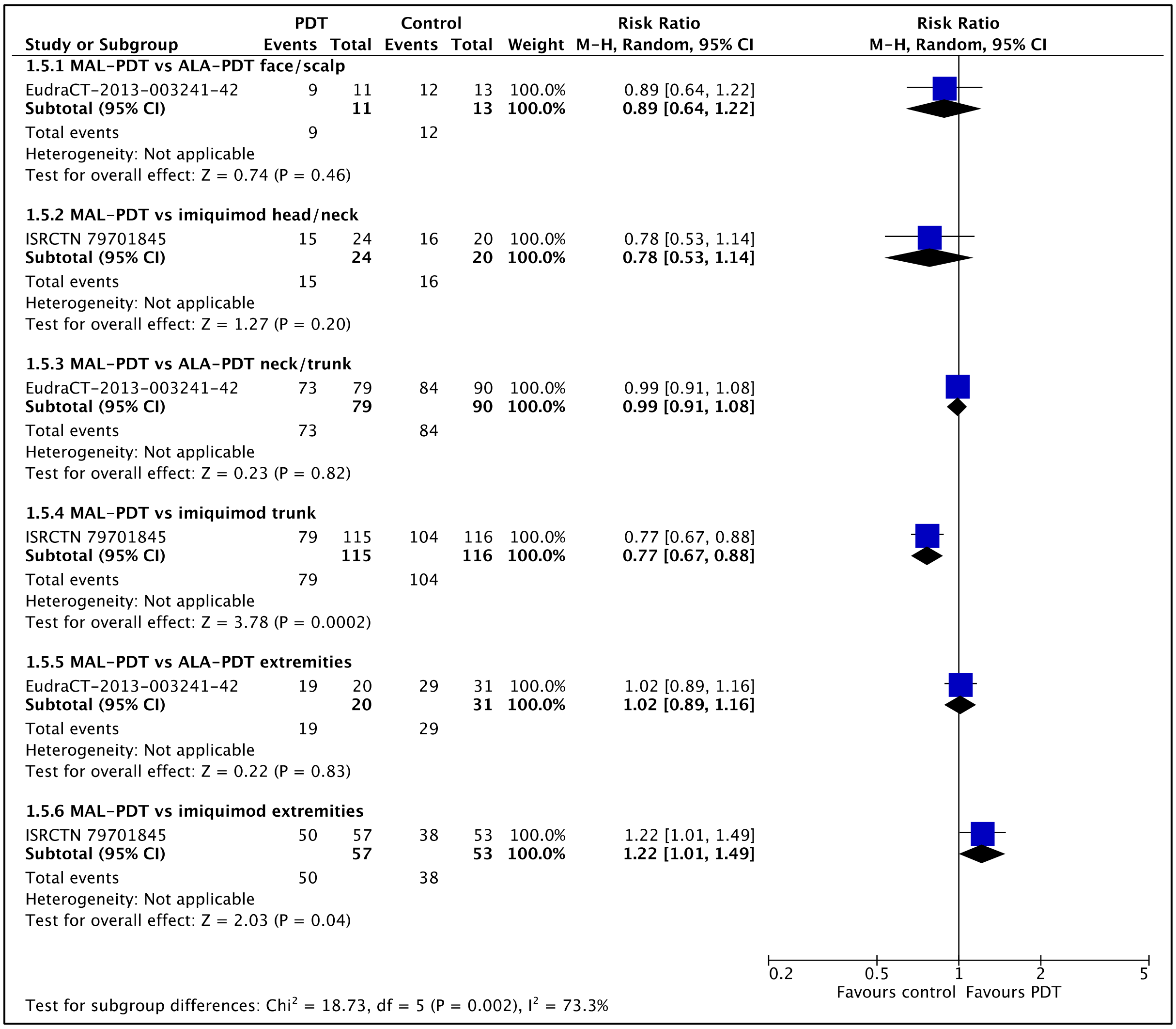

Supplementary figure 4: meta-analysis of response at 12 months by control type and location of lesion. Data are risk ratios with 95% CIs.

Study or Subgroup	MAL-PDT Events	MAL-PDT Total	Control Events	Control Total	Weight	Risk Ratio M-H, Random, 95% CI
**2.3.1 MAL-PDT vs placebo face/scalp**						
Morton 2006	2	27	1	2	100.0%	0.15 [0.02, 1.01]
**Subtotal (95% CI)**		**27**		**2**	**100.0%**	**0.15 [0.02, 1.01]**
Total events	2		1			
Heterogeneity: Not applicable						
Test for overall effect: Z = 1.95 (P = 0.05)						
**2.3.2 MAL-PDT vs cryotherapy face/scalp**						
Morton 2006	2	27	6	17	100.0%	0.21 [0.05, 0.92]
**Subtotal (95% CI)**		**27**		**17**	**100.0%**	**0.21 [0.05, 0.92]**
Total events	2		6			
Heterogeneity: Not applicable						
Test for overall effect: Z = 2.07 (P = 0.04)						
**2.3.3 MAL-PDT vs surgery face/scalp**						
Szeimie 2008	4	15	0	4	100.0%	2.81 [0.18, 43.72]
**Subtotal (95% CI)**		**15**		**4**	**100.0%**	**2.81 [0.18, 43.72]**
Total events	4		0			
Heterogeneity: Not applicable						
Test for overall effect: Z = 0.74 (P = 0.46)						
**2.3.4 MAL-PDT vs fluorouracil face/sclap**						
Morton 2006	2	27	0	5	100.0%	1.07 [0.06, 19.58]
**Subtotal (95% CI)**		**27**		**5**	**100.0%**	**1.07 [0.06, 19.58]**
Total events	2		0			
Heterogeneity: Not applicable						
Test for overall effect: Z = 0.05 (P = 0.96)						
**2.3.5 MAL-PDT vs ALA-PDT face/scalp**						
EudraCT-2013-003241-42	2	11	1	13	100.0%	2.36 [0.25, 22.70]
**Subtotal (95% CI)**		**11**		**13**	**100.0%**	**2.36 [0.25, 22.70]**
Total events	2		1			
Heterogeneity: Not applicable						
Test for overall effect: Z = 0.75 (P = 0.46)						
**2.3.6 MAL-PDT vs imiquimod head/neck**						
ISRCTN 79701845	9	24	4	20	100.0%	1.88 [0.68, 5.19]
**Subtotal (95% CI)**		**24**		**20**	**100.0%**	**1.88 [0.68, 5.19]**
Total events	9		4			
Heterogeneity: Not applicable						
Test for overall effect: Z = 1.21 (P = 0.23)						
**2.3.7 MAL-PDT vs placebo neck/trunk**						
Morton 2006	2	13	1	1	100.0%	0.24 [0.06, 0.95]
**Subtotal (95% CI)**		**13**		**1**	**100.0%**	**0.24 [0.06, 0.95]**
Total events	2		1			
Heterogeneity: Not applicable						
Test for overall effect: Z = 2.04 (P = 0.04)						
**2.3.8 MAL-PDT vs surgery neck/trunk**						
Szeimie 2008	3	69	0	83	100.0%	8.40 [0.44, 159.88]
**Subtotal (95% CI)**		**69**		**83**	**100.0%**	**8.40 [0.44, 159.88]**
Total events	3		0			
Heterogeneity: Not applicable						
Test for overall effect: Z = 1.42 (P = 0.16)						
**2.3.9 MAL-PDT vs cryotherapy neck/trunk**						
Morton 2006	2	13	1	13	100.0%	2.00 [0.21, 19.44]
**Subtotal (95% CI)**		**13**		**13**	**100.0%**	**2.00 [0.21, 19.44]**
Total events	2		1			
Heterogeneity: Not applicable						
Test for overall effect: Z = 0.60 (P = 0.55)						
**2.3.10 MAL-PDT vs fluorouracil neck/trunk**						
Morton 2006	2	13	1	2	100.0%	0.31 [0.05, 2.02]
**Subtotal (95% CI)**		**13**		**2**	**100.0%**	**0.31 [0.05, 2.02]**
Total events	2		1			
Heterogeneity: Not applicable						
Test for overall effect: Z = 1.23 (P = 0.22)						
**2.3.11 MAL-PDT vs ALA-PDT neck/trunk**						
EudraCT-2013-003241-42	6	79	6	90	100.0%	1.14 [0.38, 3.39]
**Subtotal (95% CI)**		**79**		**90**	**100.0%**	**1.14 [0.38, 3.39]**
Total events	6		6			
Heterogeneity: Not applicable						
Test for overall effect: Z = 0.23 (P = 0.81)						
**2.3.12 MAL-PDT vs imiquimod trunk**						
ISRCTN 79701845	36	115	12	116	100.0%	3.03 [1.66, 5.52]
**Subtotal (95% CI)**		**115**		**116**	**100.0%**	**3.03 [1.66, 5.52]**
Total events	36		12			
Heterogeneity: Not applicable						
Test for overall effect: Z = 3.62 (P = 0.0003)						
**2.3.13 MAL-PDT vs placebo extremities**						
Morton 2006	11	63	0	1	100.0%	0.72 [0.06, 8.39]
**Subtotal (95% CI)**		**63**		**1**	**100.0%**	**0.72 [0.06, 8.39]**
Total events	11		0			
Heterogeneity: Not applicable						
Test for overall effect: Z = 0.26 (P = 0.79)						
**2.3.14 MAL-PDT vs surgery extremities**						
Szeimie 2008	4	34	0	31	100.0%	8.23 [0.46, 146.90]
**Subtotal (95% CI)**		**34**		**31**	**100.0%**	**8.23 [0.46, 146.90]**
Total events	4		0			
Heterogeneity: Not applicable						
Test for overall effect: Z = 1.43 (P = 0.15)						
**2.3.15 MAL-PDT vs imiquimod extremities**						
ISRCTN 79701845	7	57	15	53	100.0%	0.43 [0.19, 0.98]
**Subtotal (95% CI)**		**57**		**53**	**100.0%**	**0.43 [0.19, 0.98]**
Total events	7		15			
Heterogeneity: Not applicable						
Test for overall effect: Z = 2.01 (P = 0.04)						
**2.3.16 MAL-PDT vs cryotherapy extremities**						
Morton 2006	11	63	8	43	100.0%	0.94 [0.41, 2.14]
**Subtotal (95% CI)**		**63**		**43**	**100.0%**	**0.94 [0.41, 2.14]**
Total events	11		8			
Heterogeneity: Not applicable						
Test for overall effect: Z = 0.15 (P = 0.88)						
**2.3.17 MAL-PDT vs flurouracil extremities**						
Morton 2006	11	63	3	17	100.0%	0.99 [0.31, 3.15]
**Subtotal (95% CI)**		**63**		**17**	**100.0%**	**0.99 [0.31, 3.15]**
Total events	11		3			
Heterogeneity: Not applicable						
Test for overall effect: Z = 0.02 (P = 0.99)						
**2.3.18 MAL-PDT vs ALA-PDT extremities**						
EudraCT-2013-003241-42	1	20	2	31	100.0%	0.78 [0.08, 8.00]
**Subtotal (95% CI)**		**20**		**31**	**100.0%**	**0.78 [0.08, 8.00]**
Total events	1		2			
Heterogeneity: Not applicable						
Test for overall effect: Z = 0.21 (P = 0.83)						

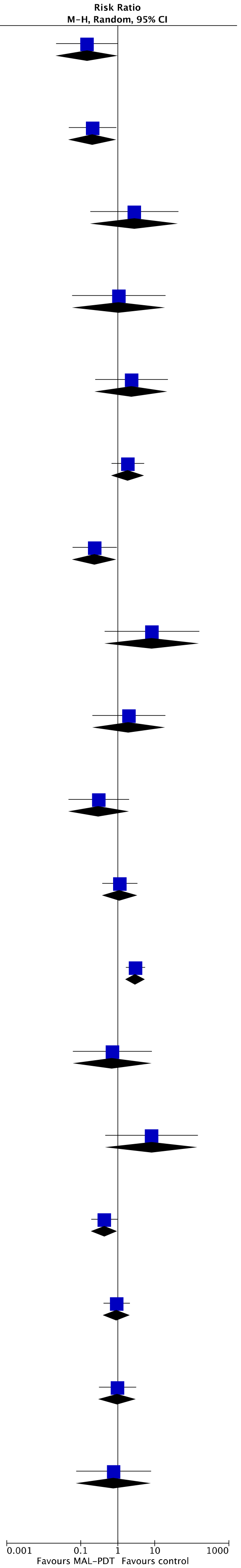

Test for subgroup differences: $Chi^2 = 37.68$, df = 17 (P = 0.003), $I^2 = 54.9\%$

Supplementary figure 5: meta-analysis of recurrence at 12 months by control type and location of lesion. Data are risk ratios with 95% CIs.

Study or Subgroup	PDT Mean	PDT SD	PDT Total	Control Mean	Control SD	Control Total	Weight	Mean Difference IV, Random, 95% CI
**4.3.1 MAL-PDT vs HAL-PDT**								
NCT02367547	1.7	2.28	27	1.5	2.16	24	100.0%	0.20 [−1.02, 1.42]
**Subtotal (95% CI)**			**27**			**24**	**100.0%**	**0.20 [−1.02, 1.42]**
Heterogeneity: Not applicable								
Test for overall effect: Z = 0.32 (P = 0.75)								
**4.3.2 MAL-PDT vs YAG-AFL-PDT**								
Ko 2013	4.3	1.767	21	4.857	2.035	21	28.5%	−0.56 [−1.71, 0.60]
NCT02018679	4.222	1.865	19	4.632	1.257	20	37.6%	−0.41 [−1.41, 0.59]
NCT02666534	5.7	1.7	19	6	1.7	21	34.0%	−0.30 [−1.35, 0.75]
**Subtotal (95% CI)**			**59**			**62**	**100.0%**	**−0.41 [−1.03, 0.20]**
Heterogeneity: $Tau^2 = 0.00$; $Chi^2 = 0.10$, df = 2 (P = 0.95); $I^2 = 0\%$								
Test for overall effect: Z = 1.32 (P = 0.19)								
**4.3.3 MAL-PDT vs ALA-PDT**								
EudraCT-2013-003241-42	4.1	2.7	142	4.5	2.7	138	48.0%	−0.40 [−1.03, 0.23]
NCT01491711	2.48	2.54	73	3.36	2.57	80	34.0%	−0.88 [−1.69, −0.07]
NCT02367547	1.7	2.28	27	1.4	2.16	26	18.1%	0.30 [−0.90, 1.50]
**Subtotal (95% CI)**			**242**			**244**	**100.0%**	**−0.44 [−0.98, 0.11]**
Heterogeneity: $Tau^2 = 0.06$; $Chi^2 = 2.62$, df = 2 (P = 0.27); $I^2 = 24\%$								
Test for overall effect: Z = 1.57 (P = 0.12)								
**4.3.4 ALA-PDT vs surgery**								
Berroeta 2007	5	2.28	21	0	2.16	19	100.0%	5.00 [3.62, 6.38]
**Subtotal (95% CI)**			**21**			**19**	**100.0%**	**5.00 [3.62, 6.38]**
Heterogeneity: Not applicable								
Test for overall effect: Z = 7.12 (P < 0.00001)								
**4.3.5 ALA-PDT vs cryotherapy**								
Wang 2001	4.3	3.1	44	3.2	2.7	39	100.0%	1.10 [−0.15, 2.35]
**Subtotal (95% CI)**			**44**			**39**	**100.0%**	**1.10 [−0.15, 2.35]**
Heterogeneity: Not applicable								
Test for overall effect: Z = 1.73 (P = 0.08)								

Test for subgroup differences: $Chi^2 = 57.58$, df = 4 (P < 0.00001), $I^2 = 93.1\%$

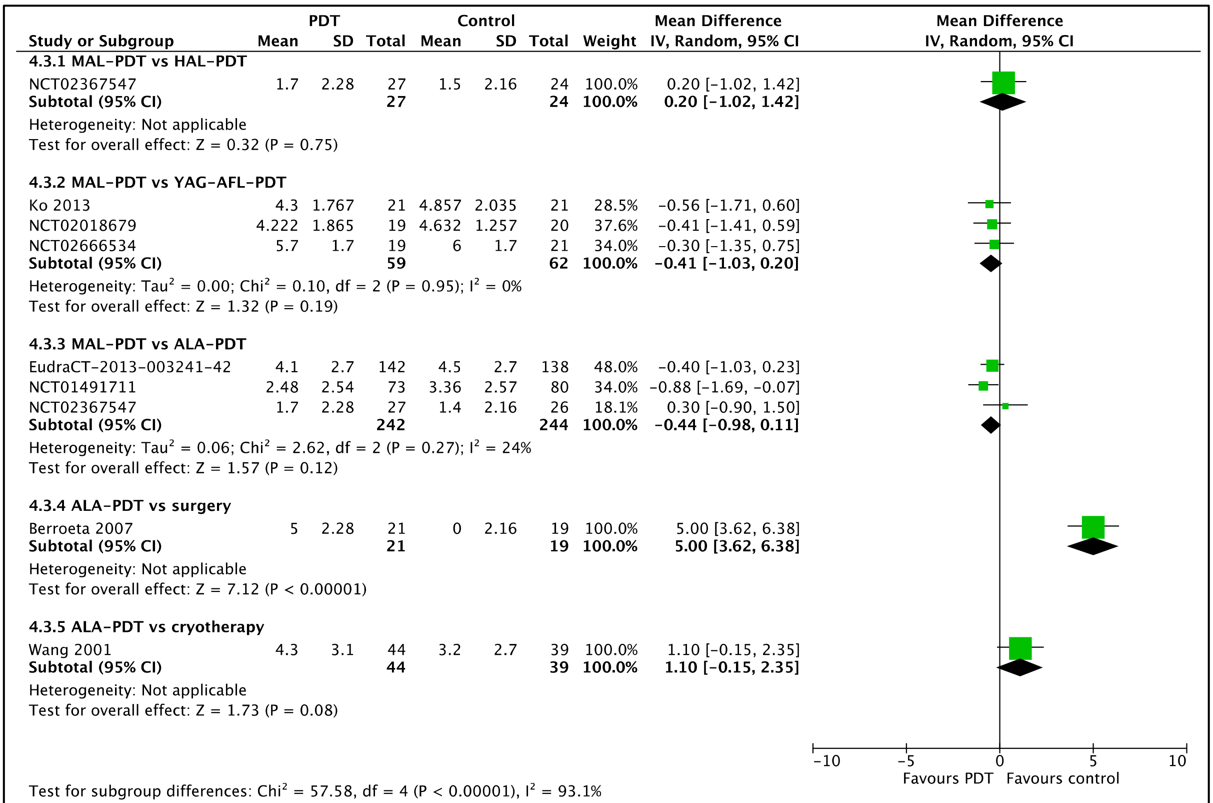

Supplementary figure 6: meta-analysis of difference in peak pain score by control type. Data were calculated using an inverse variance, random effects model. Effect sizes are given as mean differences with 95% CIs.

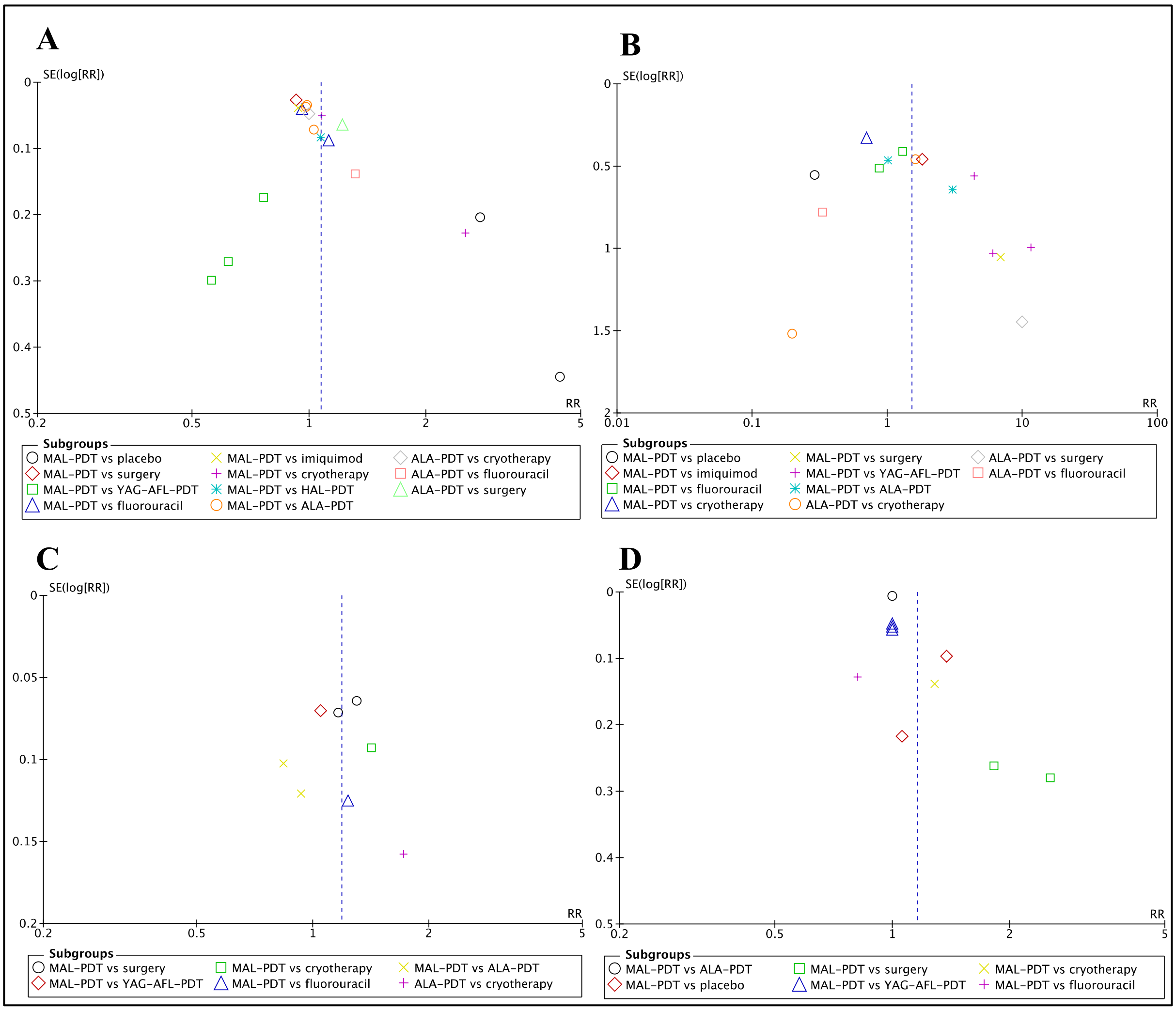

Supplementary figure 7: funnel plots of response (3 m), recurrence (12 m), cosmetic outcomes (12 m), and adverse events. The risk ratios (RR) are plotted again the standard error of the logRR.

Study or Subgroup	PDT Events	PDT Total	Control Events	Control Total	Weight	Risk Ratio M–H, Random, 95% CI
**1.6.1 Industry funded**						
Foley 2009	55	75	20	75	34.5%	2.75 [1.84, 4.10]
Morton 2006	103	111	24	29	35.8%	1.12 [0.94, 1.33]
Morton 2006	103	111	4	19	29.6%	4.41 [1.84, 10.54]
**Subtotal (95% CI)**		**297**		**123**	**100.0%**	**2.29 [0.75, 7.03]**
Total events	261		48			
Heterogeneity: Tau² = 0.90; Chi² = 46.20, df = 2 (P < 0.00001); I² = 96%						
Test for overall effect: Z = 1.45 (P = 0.15)						
**1.6.2 Government/university funded**						
ISRCTN 79701845	165	196	174	198	39.5%	0.96 [0.88, 1.04]
Mosterd 2008	78	83	68	88	36.3%	1.22 [1.07, 1.38]
Salim 2003	29	33	22	33	24.2%	1.32 [1.00, 1.73]
**Subtotal (95% CI)**		**312**		**319**	**100.0%**	**1.13 [0.92, 1.39]**
Total events	272		264			
Heterogeneity: Tau² = 0.03; Chi² = 13.07, df = 2 (P = 0.001); I² = 85%						
Test for overall effect: Z = 1.15 (P = 0.25)						
Test for subgroup differences: Chi² = 1.49, df = 1 (P = 0.22), I² = 32.9%						

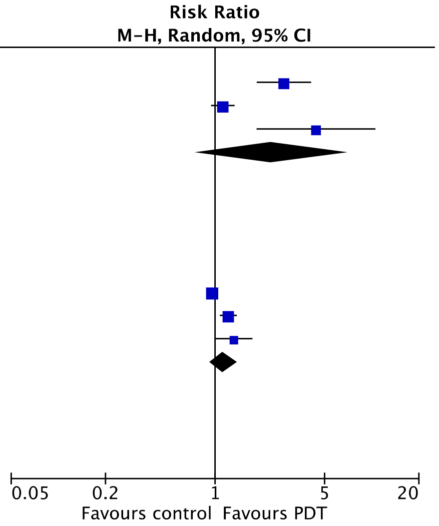

Supplementary figure 8: subgroup meta-analysis of response at 3 months by industry funding. Data are risk ratios with 95% confidence intervals.
